# Omphalocele and biliary atresia: chance or causality. A case report

**DOI:** 10.31744/einstein_journal/2022RC0072

**Published:** 2022-09-19

**Authors:** Julia Amim Rosa, Ana Maria Rossignolli Pinto, Juliana Zoboli Del Bigio, Larissa Barbosa Lima, Marcos Marques da Silva, Rafaela Braga Cabrera Mano, Mário Cícero Falcão

**Affiliations:** 1 Hospital das Clínicas Faculdade de Medicina Universidade de São Paulo São Paulo SP Brazil Instituto da Criança, Hospital das Clínicas, Faculdade de Medicina, Universidade de São Paulo, São Paulo, SP, Brazil.

**Keywords:** Congenital abnormalities, Biliary atresia, Hernia, umbilical, Jaundice, Infant, newborn

## Abstract

To relate omphalocele and biliary atresia and investigate possible embryological correlations that justify the simultaneous occurrence. A female preterm newborn diagnosed as omphalocele; cesarean delivery, weight 2,500g, 46 XX karyotype. Initially, the newborn remained fasting and on parenteral nutrition, and enteral diet was introduced later, with good acceptance. On the 12^th^ day of life, the newborn presented direct hyperbilirubinemia, increased levels of liver enzymes and fecal acholia, with a presumptive diagnosis of biliary atresia. However, the ultrasound was inconclusive, due to anatomical changes resulting from omphalocele. A surgical approach was chosen on the 37^th^ day of life aiming to confirm diagnosis of biliary atresia and to repair omphalocele. During the surgical procedure, structural alterations compatible with biliary atresia were observed, later confirmed by pathological examination; a hepatoportoenterostomy was performed and the omphalocele was corrected. She evolved well in the postoperative period, with a decrease in direct bilirubin and liver enzymes, as well as resolution of fecal acholia, and was discharged in good clinical condition. This is a bizarre and extremely rare association, but the prognosis may be good when an early diagnosis is made and surgery performed, besides support and clinical management to prevent complications in the perioperative period. Although the pathogenesis of the diseases has not been fully defined yet, there is, to date, no direct relation between them. The association between omphalocele and biliary atresia is extremely uncommon, with only two published cases.

## INTRODUCTION

Omphalocele is a midline abdominal wall defect of variable size at the base of the umbilical cord. The defect is covered by a three-layer membranous sac, consisting of amnion, Wharton gel and peritoneum. Umbilical cord vessels are inserted into the apex of the sac which normally contains herniated abdominal content.^(1)^

In addition to intestinal loops, the hernia sac may contain part of the liver, stomach, and spleen. Omphalocele can be classified as smaller (abdominal orifice up to 5cm) or giant (greater than 5cm), usually with liver in the hernia sac. It may be isolated or associated with other defects, including chromosome alterations (20% of cases), such as trisomy 13, 18 and 21, Beckwith-Wiedemann syndrome (macroglossia, gigantism, hypoglycemia and omphalocele), tetralogy of Fallot, pentalogy of Cantrell (defects in the sternum, pericardium, heart, abdominal wall, and diaphragm). Cardiac abnormalities are the most frequent (45%). Other genital, renal, and gastrointestinal abnormalities may also be present.^(2)^

On the other hand, biliary atresia is a progressive, idiopathic, fibro-obliterative extra hepatic biliary tree disease, which presents as biliary obstruction exclusively in the neonatal period.^(1)^ Although the incidence is low (approximately 1 in 10,000 to 20,000 live births), biliary atresia is a common cause of neonatal jaundice, with indications for surgery and one of the most frequent reasons for liver transplantation in children.^(3)^

The aim of this case report is to describe the association of omphalocele and biliary atresia and investigate possible embryological correlations that justifies the simultaneous occurrence.

## CASE REPORT

**Figure 1:**
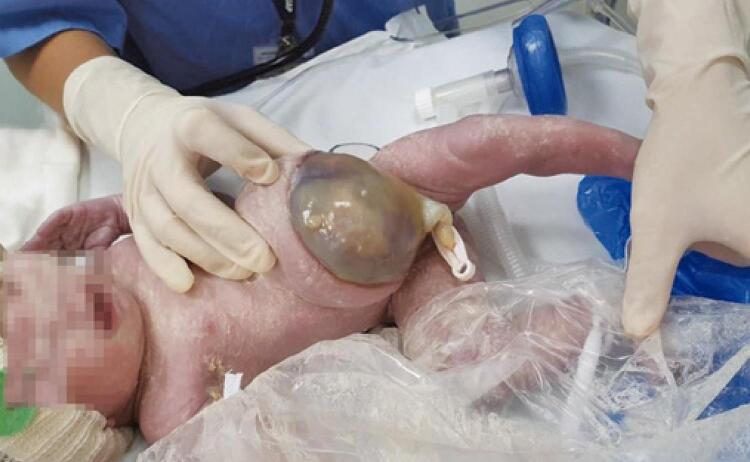
Omphalocele at birth

A female preterm newborn diagnosed as omphalocele (Figure 1); cesarean delivery, weight 2,500g, 46 XX karyotype. Initially, the newborn remained fasting and on parenteral nutrition, and enteral diet was later introduced and well accepted. The newborn presented late neonatal sepsis with a urinary source, at seven days of life (*Enterobacter cloacae* complex) and was treated with antibiotic therapy guided by antibiogram. On the 12^th^ day of life, the newborn presented direct hyperbilirubinemia, elevation of liver enzymes and fecal acholia, with a presumptive diagnosis of biliary atresia. However, the ultrasound was inconclusive due to anatomical changes resulting from omphalocele.

A surgical approach was chosen on the 37^th^ day of life, aiming to confirm the diagnosis of biliary atresia and correct the omphalocele.

There are three treatment options for omphalocele: primary closure, stepwise closure with silo placement, or closure after epithelialization. In small defects (2cm), primary closure is usually possible. In moderate to large defects (2 to 9cm), it is preferred to place a silo, for subsequent closure, after growth of the abdominal wall. When the defect is too large (>10cm) or before respiratory dysfunction, topical sclerosing agents can be applied temporarily until definitive treatment is possible.^(4)^

In the case described, although the defect was moderate (4cm), primary closure was chosen. This method reduces the risk of bacterial contamination, sepsis, acidosis, hypothermia, but can be difficult when abdominal cavity size is limited, resulting in excessive increase in intra-abdominal pressure.

**Figure 2:**
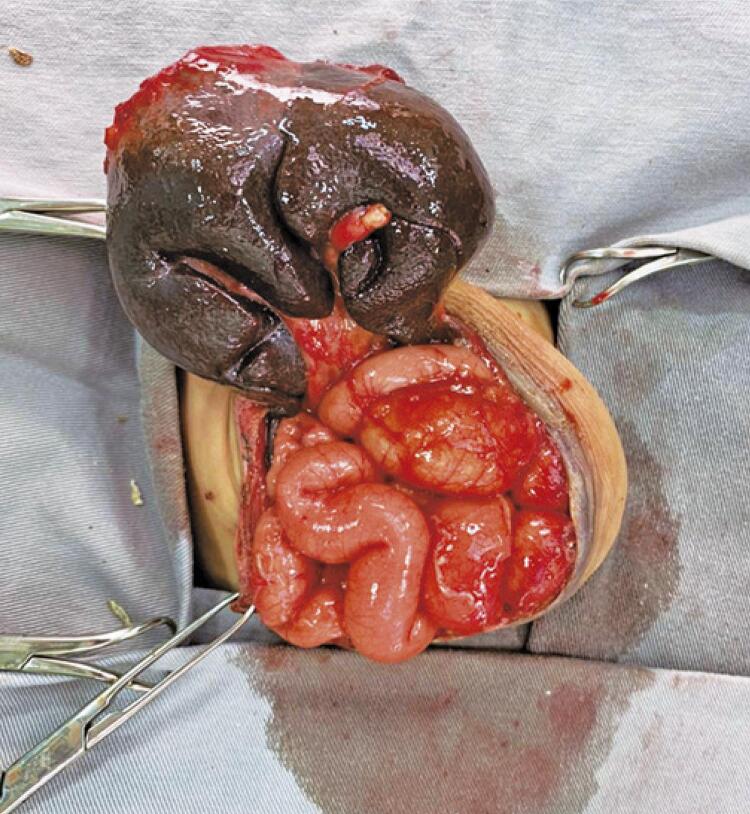
Kasai surgery (hepatoportoenterostomy)

For the correction of bile duct atresia, the classic Kasai technique, also called Kasai portoenterostomy, was used. The aim is to make a connection of the small intestine to the site of greater bile accumulation in the liver (Figure 2). This procedure allows optimizing the drainage of intrahepatic bile ducts directly into the small intestine.^(5)^

During the surgical procedure, structural alterations compatible with biliary atresia were observed, later confirmed by the pathological examination (expansion of the gate spaces by ductal proliferation, inflammatory infiltrate; bile plugs in the bile ducts; formation of port-to-port bridges; ballooning and giant transformation of hepatocytes).^(5)^ A hepatoportoenterostomy was performed and the omphalocele corrected.

The neonate evolved well in the postoperative period, with a decrease in direct bilirubin and liver enzymes, as well as resolution of fecal acholia; she was discharged in good clinical conditions. Other results in the immediate postoperative period showed hyponatremia (serum sodium =131mEq/l), mild acidemia (pH=7.19), elevation of blood lactate (from 18 to 29mmol/l), renal function within normal range, evaluated by diuresis and serum urea and creatinine. The other laboratory tests (complete blood count, serum potassium, calcium, magnesium, and phosphorus) showed no alterations. Serum sodium, pH, and lactate were normalized with adequate fluid and electrolyte support. Weight at discharge was 2,965g (she gained 33g/kg/day).

The project was approved by the Ethics Committee of the Department of Pediatrics and the Research Project Analysis Committee of *Hospital das Clínicas da Faculdade de Medicina da Universidade de São Paulo* (HCFMUSP), CAAE: 54973621.1.0000.0068; *#* 5.207.780).

## DISCUSSION

Fetal abdominal wall defects result from organogenesis disturbances during the embryonic period.^(3)^ The pathogenesis of omphalocele has not been fully established, and there is no consensus in the literature. Although there are several theories, the embryonic dysplasia theory proposes that early germinal disc defects lead to a series of malformations often seen with the amniotic band sequence. The embryonic dysplasia theory proposes that early germinal disc defects lead to a series of malformations often seen with the amniotic band sequence.^(6)^

Omphalocele may be associated with other conditions, such as structural abnormalities (gastrointestinal abnormalities, heart defects, genitourinary abnormalities, orofacial clefts, neural tube defects, and diaphragmatic defects), chromosomal abnormalities (high prevalence of aneuploidy, particularly trisomy 18 or 13), genetic syndromes (Beckwith-Wiedemann syndrome with midline malformations), polyhydramnios, and uterine growth restriction.^(7,8)^

The exact cause of bile duct atresia is unknown, and several mechanisms have been studied. The possible etiologies include viral (a specific virus has not been implicated yet; however, a study reported infants who are positive for cytomegalovirus immunoglobulin M reduced jaundice clearance after Kasai hepatoportoenterostomy); toxic (a new isoflavonoid toxin was isolated from the plant *Dysphania,* which caused severe damage to extra hepatic biliary tree in an animal model); genetic (several genetic mutations under investigation - CFC1, PKD1L1, FOXA2, EFEMP1, ADD3 - but without a well-established causal factor, suggesting a role in susceptibility to disease development), and immunological (immune dysregulation, either as a primary disturbance or as a result from infectious or genetic triggers, has been implicated in several studies).^(9-11)^

Children with biliary atresia may be grouped into three categories: biliary atresia without other anomalies or malformations (70-80% of cases, jaundice is developed in the first months of life and the stools become progressively acholic); biliary atresia associated to laterality malformations (also known as splenic biliary atresia or “embryonic” biliary atresia, laterality malformations include situs inversus, asplenia or polysplenia, malrotation, interruption of inferior vena cava and cardiac anomalies); and biliary atresia associated to other congenital malformations, including intestinal atresia, imperforate anus, kidney anomalies and/ or heart malformations.^(9-11)^

The present case is the third reported in the literature showing a rare and extremely unusual association of omphalocele and biliary atresia. The first description of this association was reported in 1971. The authors described a full-term newborn with a small omphalocele. Jaundice was observed on the fourth day of life. The neonate evolved to death on the 10^th^ day of life, and diagnosis of biliary atresia was a finding in necropsy.^(12)^ The second case was described by Pereira et al., in 2005. An 18-day-old newborn with omphalocele, biliary atresia and accessory liver lobe was successfully submitted, at 44 days of life, to portoenterostomy and repair of omphalocele. After surgery, the child had good outcomes in a 15-year follow-up.^(13)^

## CONCLUSION

The aim of this case report was to describe a newborn with two conditions (omphalocele and biliary atresia) in the neonatal period, and to investigate possible embryological correlations that justify their simultaneous occurrence. The pathogenesis of both diseases has not been well defined yet, and there is no direct correlation between them. It is a bizarre and extremely rare association but may have a good prognosis when early diagnosis is made and surgery performed, besides an adequate clinical support and management to prevent perioperative complications.
